# Distributed embodiment of metaphorical *hope* in hand, head, and eyebrow gestures

**DOI:** 10.3389/fpsyg.2023.1139881

**Published:** 2023-03-22

**Authors:** Omid Khatin-Zadeh, Danyal Farsani, Jiehui Hu, Mirko Farina, Hassan Banaruee, Fernando Marmolejo-Ramos

**Affiliations:** ^1^School of Foreign Languages, University of Electronic Science and Technology of China, Chengdu, China; ^2^Norwegian University of Science and Technology, Trondheim, Norway; ^3^Faculty of Humanities and Social Sciences, Human Machine Interaction Lab, Innopolis University, Innopolis, Russia; ^4^Department of English, American, and Celtic Studies, The University of Bonn, Bonn, Germany; ^5^University of South Australia, Adelaide, SA, Australia

**Keywords:** distributed embodiment, gesture, hope, metaphoric conceptualization, semiotics

## Abstract

This study aimed to examine the embodied conceptualization of hope through metaphors. We asked a group of participants to discuss their hopes in a semi-structured interview. We examined the types of hand, head, and eyebrow gestures produced when they were talking about their future hopes. The obtained results showed that when participants talked about their future hopes, they mainly used forward hand gestures, rightward head gestures, and upward eyebrow gestures. Based on these results, it is suggested that various semantic components and emotional associations of hope are metaphorically embodied in different manners in various parts of the body. The future aspect of hope is conceptualized as a forward movement and is embodied as a forward hand gesture. The good or positive emotional aspect associated with future hopes is metaphorically conceptualized as a rightward head gesture or an upward eyebrow gesture. We call this process distributed embodiment of a metaphorical concept. Our proposal is supported by the findings of past studies that have found future is metaphorically embodied as something in front of us (or forward movement), and good is metaphorically embodied as upper space (or upward movement) or right side (or rightward movement).

## 1. Introduction

After the emergence of the conceptual metaphor theory (Lakoff and Johnson, 1980) and the affirmation of research on embodiment in linguistics (e.g., Gallese and Lakoff, 2005), the process of embodied conceptualization through metaphors has been the subject of a large body of theoretical and empirical works in cognitive linguistics, cognitive psychology, and related fields (e.g., Gibbs, 2006; Ritchie, 2008; Johnson, 2015; Harrison and Fleming, 2019; Li et al., 2022; Khatin-Zadeh et al., 2022a,b,c; Khatin-Zadeh, 2023). According to Lakoff and Johnson (1980), metaphoric conceptualization is a process through which we understand one thing (target of the metaphor) in terms of another thing (base of the metaphor), usually a less familiar abstract concept in terms of a more familiar concrete concept. Gallese and Lakoff (2005) argue that the same cognitive resources and neural networks that are activated/recruited to process the base of a metaphor are activated/recruited to process the target of the metaphor. Therefore, for example, when we metaphorically understand the target concept of *happiness* in terms of the base concept of *up* (*happiness is up*), the same cognitive resources and neural networks that are involved in the processing of the spatial concept of *up* are employed to process the concept of *happiness*. Another heavily cited and discussed example is the metaphorical phrase *grasp an idea*. In this metaphor, understanding an idea (target of the metaphor) is described in terms of grasping an object (base of the metaphor). Gallese and Lakoff (2005) argue that the same sensorimotor networks that are involved in the action of grasping an object are activated when the individual processes this metaphor. Therefore, in this metaphor, understanding an idea is conceptualized and embodied as the action of grasping an object. In other words, Gallese and Lakoff (2005) hold that the action of grasping an object is simulated in the mind when this metaphor is processed.

Several other studies provided evidence that supports the assumptions of the conceptual metaphor. For instance, Wilson and Gibbs (2007), examined a group of participants’ understanding of a set of metaphors when a real or imagined body movement preceded the metaphors. In their experiment, participants performed or imagined a body movement related to the movement referred to in the subsequent metaphor. For example, participants performed or imagined a pushing movement and then read the metaphorical phrase *push an argument*. The results suggested that participants’ comprehension of metaphors was enhanced when metaphors were preceded by performing or imagining a body movement that was referred to in the subsequent metaphor (base of the subsequent metaphor). When mismatched body movements preceded metaphors, participants were less successful in interpreting the subsequent metaphor. These results support the idea that processing a metaphor that describes a target concept in terms of a body movement involves a simulation of that body movement. Building and expanding on this early work, Horchak et al. (2014) conducted two experiments to examine participants’ comprehension of metaphorical sentences that described abstract concepts in terms of body movements. In the first experiment, participants read a text that described an individual making metaphorical forward movements while participants’ body movements were manipulated to be congruent or incongruent with the movement referred to in the metaphor. In the second experiment, participants read the text while their body postures were manipulated to be congruent or incongruent with the movement referred to in the metaphor. A set of follow-up questions measured participants’ accuracy as well as speed of discourse comprehension. The results showed that action simulation deeply affected participants’ comprehension. This again supports one of the primary assumptions of Gallese and Lakoff’s (2005) theory; namely, when a metaphor that describes a concept in terms of a body movement is processed, that body movement is simulated in the individual’s mind.

Interestingly, results of other studies have shown that embodied simulation of metaphorical concepts occurs even among young children. Özçalışkan (2007) demonstrated that by age 5, children begin to produce metaphorical gestures that are aligned with the target domains of metaphors. For example, when using the metaphor *time is running*, they use a gesture that shows the action of running. This -once again- suggests that the action of running is simulated when the metaphor *time is running* is produced by the child.

In our study, taking all these previous works as an important source of inspiration, we decided to investigate the embodied metaphorical conceptualization of the term *hope*. The concept of *hope* is an abstract one. Like other abstract concepts, which do not have clearly perceivable referents (Borghi et al., 2018, 2019; Borghi, 2020; Dove et al., 2022; Villani et al., 2022), the term *hope* does not refer to an easily perceivable object in the concrete environment. Abstract concepts are complex and may have a variety of dimensions such as perceptual modality strength, metacognition, social metacognition, interception, emotionality, social valence, hand activation, and mouth activation (Villani et al., 2019, 2021). As an abstract concept, *hope* can have a multiple of semantic and affective associations. Hope for future achievements and good events has a wide range of semantic dimensions. We may be hoping to obtain a college degree, to become a successful businessman or a president, etc. These prospective future achievements and events are very different in various respects. Nevertheless, all of them share one critical point: we ‘hope’ for the realization of something. In our study, we specifically focused on the embodied metaphorical conceptualization of this shared point. We intended to examine how this shared point is metaphorically embodied in gestures when people talk about their future hopes. Gesture can be seen as the visible action when it is used as an utterance or as a part of an utterance (Kendon, 2004; Rosa and Farsani, 2021). Furthermore, it can be seen as “visible embodiment” (Hostetter and Alibali, 2008, p. 495) of an internal literal or metaphoric simulation (Hostetter and Alibali, 2018), as it reflects mental simulation and interfaces with thought (Calbris, 2013). In any case, gestures that are used when people talk about *hope* can be regarded as the embodied realizations of semantic aspects of this concept. To achieve the goal of our study, we asked a group of participants to talk about their future hopes in an interview setting. We examined the types of hand, head, and eyebrow gestures produced when they were talking about their future hopes. By analyzing the types and directions of gestures, we intended to find out how the term *hope* is metaphorically conceptualized in mind and embodied in gestures. In other words, we aimed to examine the distributed embodiment of this concept in hand, head, and eyebrow gestures. Some past works have examined the distribution of various aspects of meanings of motion events across words and gestures (e.g., McNeill, 1992). Some other works have explored the distribution of various components of a performative or an emotion expression across various parts of a facial expression (Poggi and Pelachaud, 1998, 2000; Poggi and D’Errico, 2010). In our study, we specifically focused on distributed embodiment of *hope* across hand, head, and eyebrow gestures. Before going on to describe the study’s methodology, we review a number of related studies that have examined the embodied conceptualization of some concepts through metaphors.

## 2. Embodied conceptualizations of concepts through metaphors

A series of studies investigated the embodied conceptualization of some concepts across various cultures (e.g., Kövecses, 1986, 2000, 2005, 2008) and the ways that concepts are realized in gestures (Caso et al., 2006; Parzuchowski and Wojciszke, 2014). One set of works focused on the embodied conceptualization of human emotions through metaphors. According to Kövecses (1990), human emotions are metaphorically conceptualized as ‘fluids in a container’, where the container is obviously the body. In his seminal work, Kövecses (1990) discussed some examples (such as *he was filled with emotion*, *I feel empty*, *he poured out his feeling to her*, and *he bottled up his emotions*) in order to illustrate his idea. Some philosophers (e.g., Prinz, 2004) also argued that emotions are deeply embodied. Others (Griffiths and Scarantino, 2005) went even further and understood emotions as distributed phenomena designed to function in a social context and arising as a form of skillful engagements with the world. Crucially, on this latter view, emotions need not to be mediated by conceptual thought and (being dynamically coupled with the environment) can be scaffolded by it both synchronically and diachronically.

On a similar vein, examining a corpus of English metaphors, Omori (2008) claimed that emotions are primarily conceptualized and embodied as a huge mass of moving water in the natural world. Ansah (2014) studied the embodied conceptualization of one particular emotion -*fear-* in English and Akan (the language of the Akan people of Ghana, spoken over much of the southern half of Ghana). She found that *fear* is metaphorically conceptualized as a variety of embodied entities in Akan and English. For example, English people conceptualize *fear* as an embodied entity by using metaphors such as *fear is a fluid in a container*, *fear is a vicious enemy*, and *fear is a tormentor*. Akan-speaking people embody *fear* by using metaphors such as *fear is fire in a container* and *fear is an opponent*. These examples show that fear, which is an abstract concept and an internal emotional state, is metaphorically conceptualized as an embodied entity in these two languages.

Studies on the embodied conceptualization through metaphor are not restricted to human emotions. A variety of concepts has been examined across various cultures. One study investigated the embodied conceptualization of *power* as a backward/forward movement in English and Chinese (Yang et al., 2021). In two experiments of this study, an action compatibility task examined the embodied conceptualization of power as a forward/backward movement. Participants were asked to categorize a set of words into two classes of powerful and powerless as quickly as possible. In the compatible condition, participants had to make a forward movement to indicate that word was powerful and a backward movement to indicate that the word was powerless. In incompatible conditions, participants had to make a backward movement to indicate that word was powerful and a forward movement to indicate that the word was powerless. The results showed that participants were faster at categorizing the words in compatible than in incompatible conditions. This suggests that the abstract concept of power can be metaphorically embodied in terms of the concrete domain of movement in the space. Another corpus-based study investigated the embodied conceptualization of *importance* in terms of the size and weight of concrete objects in Chinese and English (Yu et al., 2017). Discussing several Chinese and English metaphors used in natural discourse, researchers of this study conclude that these metaphors are based on and derived from the OBJECT image schema. They argue that this schema is abstracted from embodied sensorimotor experiences, especially our visual and tactile experiences. Another corpus-based study found that *difficulty* is metaphorically conceptualized as weight and solidity in English and Chinese (Yu and Huang, 2019).

Among the concepts that have been the subject of this line of research, *past*/*future* and *good/bad* have had special places, as many cognitive and cultural studies have investigated them. Metaphorical expressions such as *do not look back to your past* or *the new year stumbled upon us* are two examples of a large number of metaphorical expressions that show future and past are metaphorically conceptualized as something in front of us (or a forward movement) and behind us (or a backward movement) respectively (e.g., Torralbo et al., 2006; Santiago et al., 2007; Cienki and Müller, 2008; Sell and Kaschak, 2012; Rolke et al., 2013; Eikmeier et al., 2015). However, some cross-cultural differences in the metaphorical conceptualization of time have been reported. For example, two studies have found that the future is conceptualized as something behind, and the past is conceptualized as something in front of people in Aymara (Núnez and Sweetser, 2006; Moore, 2011). Several other studies have found that the past is conceptualized as *above*, and the future is conceptualized as *below* in Mandarin Chinese (e.g., Boroditsky, 2001; Boroditsky et al., 2011). Some cross-individual differences have been reported in the metaphorical conceptualization of *good*/*bad*. Casasanto (2009) argues that right-handers associate *good* (positive things) with the right side and *bad* (negative things) with the left side, while left-handers associate *good* (positive things) with the left and *bad* (negative things) with the right side (see also Kong, 2013). In other words, depending on the fluency in a specific body part, the individual may metaphorically conceptualize good/bad as something on the right or left. Furthermore, the metaphors *good is up* and *bad is down* have been discussed in many works (e.g., Lakoff and Johnson, 1999; Glenberg and Kaschak, 2002; Casasanto, 2009; Shapiro, 2011). There is some empirical evidence showing that people metaphorically associate good (or positive) things with upper space and bad (or negative) things with lower space (Crawford et al., 2006; Meier and Dionne, 2009; Casasanto and Dijkstra, 2010). All these studies suggest that even highly abstract concepts, which are apparently detached from sensorimotor experiences, are metaphorically conceptualized and embodied through our sensorimotor systems. In fact, this is a mechanism for grounding even highly abstract concepts in the concrete environment. As mentioned above, this study aimed to examine the metaphorical conceptualization of *hope*. Our main goal was to discover how *hope* is embodied in people’s gestures when they talk about things they hoped would be realized in the future.

## 3. Method

### 3.1. Participants

We randomly selected 30 undergraduate students at Chabahar Maritime University using convenience sampling. All participants were Persian native speakers. To avoid a preference for a particular group of students or a particular criterion for selecting participants, we randomly recruited a heterogeneous group of students available on the university campus. This group consisted of 15 males and 15 females aged between 18 and 23 (Mdn_age_ = 21; MAD_age_ = 1.48). All participants were right-handed. All participants of the study participated voluntarily and gave their written informed consent.

### 3.2. Materials

The data of this study were collected through face-to-face semi-structured interviews. Two cameras were used for data collection. A front camera recorded participants’ hand, head, and eyebrow gestures from a front point. This camera recorded upward, downward, rightward, and left-hand gestures of hands and head. Also, upward and downward gestures of eyebrows were recorded by this camera. A side camera recorded participants’ hand and head gestures from a side point. This camera recorded forward and backward gestures of hands and head.

### 3.3. Design and procedure

We used a quantitative experimental design to elicit gestures from participants during an interview that took around 5 min. To familiarize the participants with the interview context, we held a preparation session. To avoid the effect of observer expectancy, we did not perform any interviews with them. We just provided detailed information and instructions. Since knowing the aim of the research could affect participants’ performance in the interviews, the study’s main aim was not revealed. One of the researchers of the study conducted the interviews. In the interviews, participants had to talk about two things they hoped to achieve in the future. This was done by simple questions such as “what do you hope to achieve in future?” This was clearly explained to the participants in the preparation session. During the interviews, the interviewer limited his talk as much as possible to give the participants time to discuss their future hopes. Using a limited number of words, the interviewer just helped the participants stay focused on the main subject of the interview. The interviewer did not use any gestures during the interviews.

### 3.4. Data analysis

The sentences that were produced by participants in the interviews were transcribed. Then, the transcriptions were coded by analyzing the videos. Where the term *hope* had been used by a participant, the type of hand, head, and eyebrow gestures produced by the participant were determined by the coder. The coding of gestures for each case of using the term *hope* was recorded in the transcription. To avoid any bias in the coding of data, two independent coders who were not involved in the study coded the data. The inter-coder reliability was calculated to make sure that the process of coding had an acceptable level of reliability. For those cases that the coders had made different judgments, one of the researchers of the study made the final decision. The videos recorded by the front camera were used to code upward, downward, rightward, and leftward gestures of hand and head. Also, these videos were used to code upward and downward movements of eyebrows. The videos recorded by the side camera were used to code forward and backward gestures of hand and head.

The occurrences of hand and head movements were modeled *via* a two-parameter Zero-Inflated Poisson (ZIP) regression model by considering all variables of interest. This distribution is suitable when there is a large proportion of 0 s in the data. In the ZIP distribution, the parameter μ corresponds to the mean of the Poisson distribution, and the parameter σ represents the inflation probability at 0 (details as to the ZIP distribution can be found in Rigby et al. (2020), section 22.2.9, pp. 498–499). The resulting model was ‘counts ~ body_part + gender + direction + age’ (body_part = is a categorical variable with the levels of head and hand movements; gender = is a categorical variable with the gender of the participants [levels male and female]; direction = is a categorical variable with the levels upward [U], downward [D], leftward [L], rightward [R], forward [F], and backward [B]; age = is a continuous variable corresponding to the age of the participants). The model was additive (i.e., no interactions were considered) and had no random effects as there were no repeated measures per combination of factors’ levels. This model was examined *via* a generalized additive model for location, scale, and shape approach (GAMLSS; Stasinopoulos et al., 2018).

The ZIP regression model was further examined *via* a GAMLSS-based distributional regression tree (Schlosser et al., 2019) to facilitate interpretation of the relationship between the significant variables and their levels. Note that regression decision trees retain variables for which significant binary splits can be performed in relation to the dependent variable. For example, a variable with two levels (e.g., males and females) will be included in a decision tree if the two levels have significant differences in both parameters of their ZIP distributions (e.g., males show higher height than females in terms of the *μ* parameter of their ZIP distributions); a variable with three levels (e.g., males, females, neutral) will be included in a decision tree if there are significant binary groupings that differ in the two parameters of their ZIP distributions (e.g., males and neutral, together, show higher height than females in terms of the μ parameter of their ZIP distributions). Thus, GAMLSS-based distributional regression trees generate those partitions based on significant differences in all the parameters of the distribution of the dependent variable. In other words, GAMLSS-based regression trees reflect fully distributional differences between variables.

A second data set considering upward and downward eyebrow movements were examined *via* the model ‘counts ~ gender + direction + age’ (gender = is a categorical variable with the gender of the participants [levels male and female]; direction = is a categorical variable with the levels upward and downward; age = is a continuous variable corresponding to the age of the participants). This model was examined *via* GAMLSS with a ZIP distribution and a distribution regression tree.

Data sets and R codes are available in Figshare at: https://cutt.ly/NXU7f6i.

## 4. Results

Table 1 shows the total number of hand and head gestures according to the direction they were performed.

**Table 1:** Total number of hand and head gestures according to the physical direction they were performed.

Physical direction	Hand	Head	Total
Upward	19	24	43
Downward	4	2	6
Rightward	49	75	124
Leftward	3	4	7
Forward	96	6	102
Backward	2	2	4
Total	173	113	

**Figure 1:**
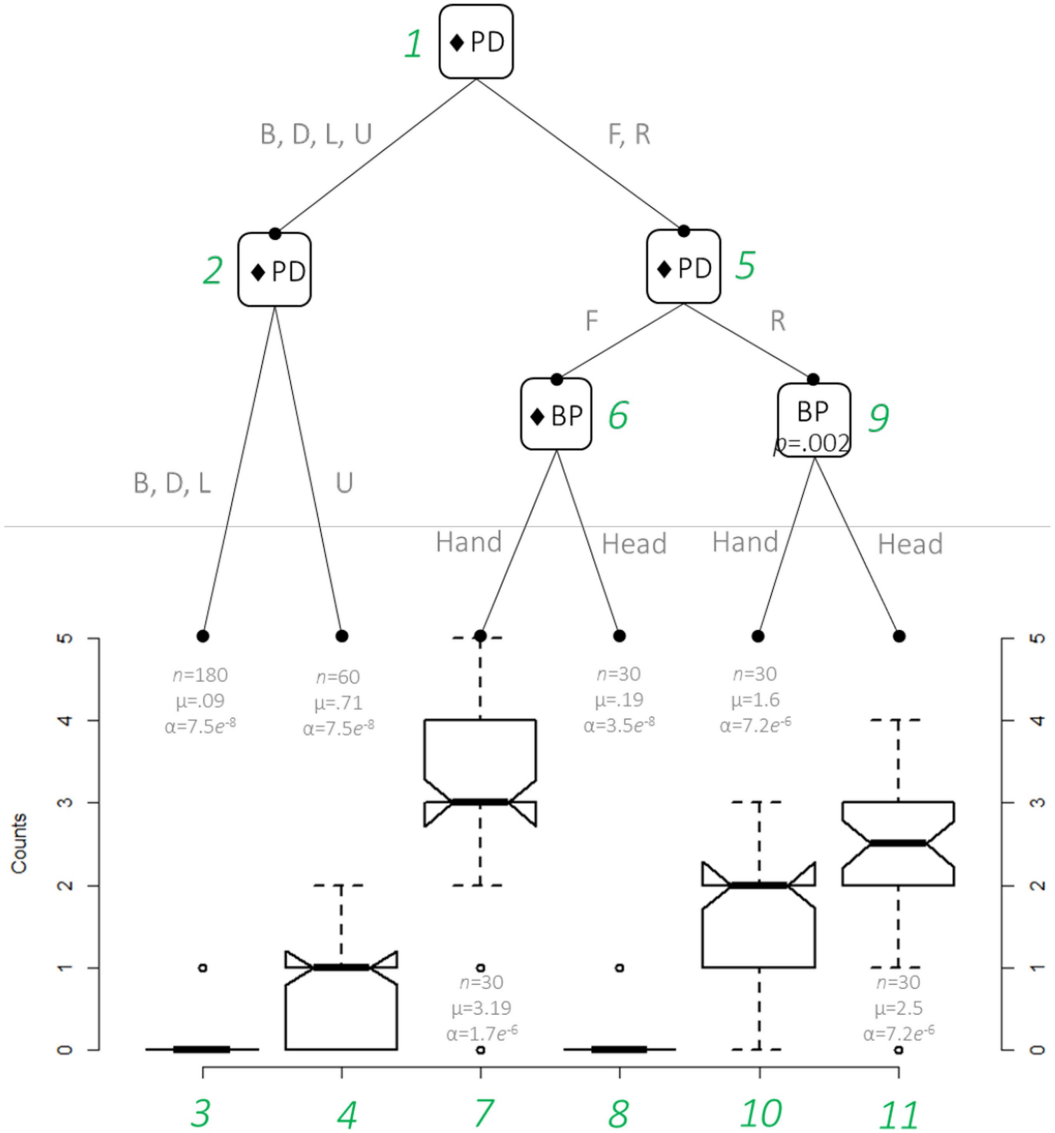
Generalized additive model for location, scale, and shape approach (GAMLSS)-based distributional regression tree showing the relationships between the significant variables body_part (BP; head and hand) and physical direction (PD; upward = U, downward = D, leftward = L, rightward = R, forward = F, and backward = B). The data were modeled with a Zero-Inflated Poisson (ZIP) distribution. ♦ = *p* < 0.001. The numbers in green show the order of the nodes. The number of observations and the ZIP distribution parameters’ values are reported, respectively, in the format ‘n = xx, mu = xx, sigma = xx.’

The results of the ZIP regression indicated that only ‘body_part’ and ‘physical direction’ were significant (see details of the ZIP regression model in the supplementary files). The differences in these two variables are evident in the counts shown in Table 1. The distributional regression tree visualizes the relationship between these two variables (see Figure 1).

The tree suggests that the most significant difference in counts was due to the physical direction of the body movements (node 1). As shown in nodes 5, 6, and 9, there were, on average, more forward, and rightward movements than other movements and there were more rightward than forward movements (*μ*_rightward_ = 2.05, *μ*_forward_ = 1.69). However, there were more forward hand than head movements (nodes 7 and 8) and more rightward head than hand movements (nodes 10 and 11). A similar logic of interpretation applies to other paths of interest.

The results of the ZIP regression model applied to the eyebrow movements data indicated that only the variable physical direction (i.e., upward vs. downward movements) was significant. On average, there were more upward (*μ* = 2.73) than downward (*μ* = 0.1) eyebrow movements.

## 5. Discussion

The results of this study may be taken to suggest that *hope* is embodied in three different directions in three different parts of the body. It is mainly embodied as a forward movement in the hands, a rightward movement in the head, and an upward movement in the eyebrows. In order to explain the variety of embodied realization of hope in various parts of the body, we need to analyze the semantic dimensions of hope. Every concept may have a variety of semantic dimensions and associations. When we talk about our hopes, we refer to a forthcoming event or achievement in the future. Therefore, one semantic association of *hope* is the future. The future is the temporal dimension of hope. Another dimension of hope is our good or positive feeling toward something that may happen in the future. In other words, the goodness and positivity of a prospective event constitute another semantic dimension of hope. When hope is embodied and realized in the form of gestures, each one of its semantic dimensions can be embodied in a certain part of the body in a particular shape. The future dimension of hope can be embodied as a forward hand gesture. As mentioned, results of some past studies have shown that the future is metaphorically embodied as something in front of us or as a forward movement (e.g., Torralbo et al., 2006; Santiago et al., 2007; Sell and Kaschak, 2012; Rolke et al., 2013; Eikmeier et al., 2015). Therefore, it can be suggested that the future, as a specific dimension of *hope*, is embodied as a forward movement, and a forward hand gesture shows this forward movement. When discussing complex concepts, ideas, or events, we may use various available resources to convey our message. We use words of speech, body gestures, physical objects in the context, background information, and many other things to express our message. This set of elements works together to convey a message. The information conveyed by the message can be distributed among these elements and expressed by all of them. In the case of *hope*, the future dimension can be assigned to a hand gesture and expressed by it in the form of a forward hand gesture. However, it must be noted that this does not mean that the future dimension is expressed solely by hand gestures. It may be expressed by other resources as well. The critical point is that hand gesture plays an active role in expressing this dimension of hope in collaboration with other potential resources that may be involved.

Having a good or positive feeling about an event or a possible achievement in the future is another dimension of *hope* that can be embodied or realized in another part of the body in the form of a gesture. As mentioned, some evidence suggests that good or positive feelings toward things are metaphorically associated with the right side of the body. However, it should be noted that some later evidence suggested that right-handers tend to associate good or positive things with the right side, while left-handers tend to associate good or positive things with the left side (Casasanto, 2009). Since all participants of our study were right-handers, it can be suggested that the good or positive dimension of *hope* has been realized in the form of rightward hand gestures.

As previously mentioned, in many metaphors, we describe *good* (or positive things) in terms of upper spatial position and *bad* (or negative things) in terms of lower spatial position. This metaphorical description can be embodied in gestures. The data of our study showed that participants used a significant number of upward eyebrow gestures while talking about their future hopes. Therefore, it can be suggested that the good or positive aspects of hope may be embodied as upward eyebrow gestures. In fact, our hopes could have a wide range of associations. These associations could be semantic or emotional. These associations may be embodied in a distributed manner among different parts of the body and realized in the form of gestures in various body parts. We call this process *distributed embodiment* (Farina and Lavazza, 2022a,b). Distributed embodiment is a process in which associations of a concept are divided into a set of interdependent components, and these components are embodied independently in various parts of the body in the form of gestures. The data of our study suggested that this process occurs when people talk about their hopes. In one of the cases observed in our study, the participant said, “I hope to get my college degree and work as a counselor in a large prestigious company.” When uttering this sentence, he used three gestures: a forward hand gesture, a rightward head gesture, and an upward eyebrow gesture. Interestingly, the participant did not use an adverb of time in this sentence. Therefore, the time he hoped to achieve this goal was not mentioned in the sentence. However, the meaning of the future is embedded in this sentence at a conceptual level, and he hoped to achieve this goal at some point in the future. This embedded but superficially-hidden part of meaning was realized as a forward hand gesture. At the same time, he had a good or positive feeling about achieving this goal in future. This feeling was realized as a rightward head gesture and an upward eyebrow gesture. Like the future dimension of *hope*, the emotional feelings associated with it are absent from the surface of the sentence and are not explicitly mentioned. They are hidden associations of this sentence at a superficial level but exist at a conceptual level. The good feeling associated with hope could be noticed in other facial expressions. However, in our study, we did not include facial expressions as a factor associated with emotional aspects of hope.

It should be noted that semantic aspects of *hope* are not limited to future and positive emotions. A sense of expectancy and passive tension (or a sense of impotence) can be other semantic associations of *hope*, since in hope one does not think one has the power to bring about one’s goal, but can only rely on external events, without having any sense of agency (Miceli and Castelfranchi, 2015). These semantic aspects of *hope* may also play a role in how this concept is embodied in hand, head, and eyebrow gestures.

Distributed embodiment is, in fact, a process for embodying the totality of a situation. When people talk about their hopes, they create an imaginary situation in which their hopes are realized. This imaginary situation could involve several semantic and contextual elements. To embody the whole situation, the elements involved in that situation must be distributed across the various parts of an embodying system. The human nervous system simulates the whole situation. While some elements involved in the situation are realized in the form of gestures, others may not. In other words, the distributed embodiment is a matter of not only embodying various semantic elements of a concept but also embodying various dimensions of a context people express.

A question raised here is, “does distributed embodiment take place for all types of concepts or just for special types?” In order to answer this question, we have to examine the associations of concepts. Among the semantic associations of a concept, the motoric associations could be an essential dimension. If a concept has solid motoric associations with various parts of the human body, the motoric associations can be activated and embodied in the form of gestures in those parts of the body when people talk about that concept (or experiences related to that concept). Another possible factor is the emotional load of the concept or experiences related to that concept. As mentioned, the results of some past studies have shown that emotional concepts may have strong motoric associations. These associations could be metonymic and metaphoric.

If a concept (or related experiences) has a significant load of semantic associations, the associations could be embodied independently in certain body parts. For example, when talking about the concept of explosion, we may show the occurrence of an explosion or its consequences by hand gestures. At the same time, we show our fear of the explosion by a backward head movement. Here, while hand gestures describe the physical dimensions of the explosion of the concept, the emotional aspect associated with the explosion (fear) is shown by a backward head gesture. It should be noted that fear is not one part of the meaning of an explosion. However, it has some association with it due to our past experiences. This was observed in the results of our study in the case of hope as a concept with some positive emotional associations. A good or positive feeling toward something that may happen in the future is associated with people’s hopes. This emotional association is embodied as an upward gesture in the eyebrows.

## 6. Limitations

Like any other empirical study, our study had some limitations that could restrict the scope of interpretation of our data. Firstly, although we did our best to create a natural setting in the interview, the context of any interview could have some kind of influence on the behavior of the interviewee. If the data of our study had been gathered in a more natural environment over an extended period of time, more accurate and reliable results could have been obtained. Secondly, the data of our study were gathered from Persian native speakers. However, there might be some cross-cultural differences between the ways that a given concept is embodied. This has been supported by evidence from studies that have investigated the embodiment of some concepts in various cultures (e.g., Kövecses, 1986, 1990, 2000, 2005; Núnez and Sweetser, 2006; Moore, 2011). If the data of our study had been gathered from a variety of cultures, more general conclusions could have been reached. Thirdly, in our study, we specifically focused on the movements of hands, head, and eyebrows. However, the process of embodiment may be realized across a wider range of body parts. For example, facial expressions constitute an important emotional dimension of the embodiment of the concept of hope. Because of lack of equipment, we did not include a wider range of dimensions associated with the embodiment of hope.

Another point that should be noted here is that we do not take a nativist approach to cognition. Although the data of our study suggest that various dimensions of the concept of hope are embodied in certain ways in various body parts, it does not mean that these embodied realizations are encoded, inborn, and phylogenetically inherited in certain body organs. Like any other abstract concept, the concept of hope is acquired over a period of time during human cognitive development and is metaphorically represented in terms of body movements. This means that various dimensions of this concept are formed and metaphorically represented in the human cognitive system. These embodied representations gradually appear in a gestural format in human gestures.

## 7. Conclusion

Results of this study suggested that when people talk about their hopes, they use a variety of metaphoric gestures in various parts of the body. The types and directions of these gestures suggest that various dimensions of hope are embodied in different ways in various body parts. More precisely, it seems that the meaning and associations of hope are divided into a set of components, which are distributed and embodied in various parts of the body. Since these components may be different in terms of nature, the types of gestures used to embody them may be different. For example, the future dimension of hope is very different from its emotional dimension. These two dimensions are metaphorically described in different manners, and the types of gestures used to embody them differ in direction, shape, and maybe some other aspects. The future dimension of hope is embodied as a forward movement, while emotional aspects associated with hope are embodied as rightward and upward gestures. Therefore, the first is embodied as a forward gesture of the hand, while the second is embodied as a rightward gesture or upward gesture of the head and eyebrows. This suggests that a coordinated embodying system can operate when *hope* is metaphorically described and embodied. The components of this embodying system work in a coordinated manner and offer an embodied representation of this concept. In this study, we specifically focused on the distributed embodiment of hope. However, this process can take place for many concepts. This is a question that can be addressed in future research projects.

## Data availability statement

The original contributions presented in the study are publicly available. This data can be found at: https://figshare.com/projects/Embodiment_of_hope/146697.

## Ethics statement

The studies involving human participants were reviewed and approved by The study was carried out according to the declaration of Helsinki (World Medical Association, 2013) and was approved by ethical committee of Chabahar Maritime University. The patients/participants provided their written informed consent to participate in this study.

## Author contributions

OK-Z wrote the first draft of the paper. DF, JH, MF, HB, and FM-R commented on it and improved the paper. All authors contributed to the article and approved the submitted version.

## Funding

This work was supported by the Norwegian University of Science and Technology.

## Conflict of interest

The authors declare that the research was conducted in the absence of any commercial or financial relationships that could be construed as a potential conflict of interest.

## Publisher’s note

All claims expressed in this article are solely those of the authors and do not necessarily represent those of their affiliated organizations, or those of the publisher, the editors and the reviewers. Any product that may be evaluated in this article, or claim that may be made by its manufacturer, is not guaranteed or endorsed by the publisher.
